# Metagenomic Analysis of Biocide-Treated Neotropical Oil Reservoir Water Unveils Microdiversity of Thermophile *Tepidiphilus*

**DOI:** 10.3389/fmicb.2021.741555

**Published:** 2021-11-01

**Authors:** Katherine Bedoya, Jhorman Niño, Julia Acero, Ronald Jaimes-Prada, Felipe Cabarcas, Juan F. Alzate

**Affiliations:** ^1^Facultad de Medicina, Centro Nacional de Secuenciación Genómica - CNSG, Sede de Investigación Universitaria - SIU, Universidad de Antioquia -UdeA, Medellín, Colombia; ^2^Centro de Innovación y Tecnología ICP, Ecopetrol S.A, Gerencia de Operaciones, Bucaramanga, Colombia; ^3^Grupo SISTEMIC, Ingeniería Electrónica, Facultad de Ingeniería, Universidad de Antioquia - UdeA, Medellín, Colombia

**Keywords:** extremophile, comparative genomics, metagenomics, *Tepidiphilus*, thermophile, biocide resistance

## Abstract

Microorganisms are capable of colonizing extreme environments like deep biosphere and oil reservoirs. The prokaryotes diversity in exploited oil reservoirs is composed of indigenous microbial communities and artificially introduced microbes. In the present work, high throughput sequencing techniques were applied to analyze the microbial community from the injected and produced water in a neotropical hyper-thermophile oil reservoir located in the Orinoquia region of Colombia, South America. *Tepidiphilus* is the dominant bacteria found in both injection and produced waters. The produced water has a higher microbial richness and exhibits a *Tepidiphilus* microdiversity. The reservoir injected water is recycled and treated with the biocides glutaraldehyde and tetrakis-hydroxymethyl-phosphonium sulfate (THPS) to reduce microbial load. This process reduces microbial richness and selects a single *Tepidiphilus* genome (*T.* sp. UDEAICP_D1) as the dominant isolate. *Thermus* and *Hydrogenobacter* were subdominants in both water systems. Phylogenomic analysis of the injection water dominant *Tepidiphilus* positioned it as an independent branch outside *T. succinatimandens* and *T. thermophilus* lineage. Comparative analysis of the *Tepidiphilus* genomes revealed several genes that might be related to the biocide-resistant phenotype and the tolerance to the stress conditions imposed inside the oil well, like RND efflux pumps and type II toxin-antitoxin systems. Comparing the abundance of *Tepidiphilus* protein-coding genes in both water systems shows that the biocide selected *Tepidiphilus* sp. UDEAICP_D1 genome has enriched genes annotated as ABC-2 type transporter, ABC transporter, Methionine biosynthesis protein MetW, Glycosyltransferases, and two-component system NarL.

## Introduction

Oil reservoirs exhibit extreme environmental conditions for microbial life such as high temperature, salinity, pressure, anoxic conditions, and presence of heavy metals (Pannekens et al., 2019; Roumagnac et al., 2020). Nevertheless, the crude oil, the aqueous phase, and solid surfaces in the well can harbor complex microbial communities with the capability to thrive in these extreme environments (Liu et al., 2018; Pannekens et al., 2019). Sulfate-reducing bacteria, nitrate-reducing bacteria, fermentative bacteria, syntrophic bacteria, methanogens, and many more microorganisms can be found in oil reservoirs (Liang et al., 2018). The structure of natural microbial communities can be explained by niche-based mechanisms (Yuan et al., 2021), and differences in the abundance of taxa reflect the differences in the environmental factors (Gao et al., 2016). Among these, temperature has been considered the highest theoretical limiting factor controlling microbial growth in petroleum reservoirs (Magot et al., 2000).

Indigenous and introduced microorganisms have several effects in petroleum reservoirs and oil exploitation (Ren et al., 2015); some of them are detrimental producing hydrogen sulfide (souring), inducing corrosion or leading to oil pipelines clogging (Okoro et al., 2016; Dolfing and Hubert, 2017; Vigneron et al., 2017). Chemical and physical treatments are widely applied to reduce the microbial load introduced in the secondary oil recovery strategies (Jurelevicius et al., 2021). In the oil industry, the biocide treatment can be applied continuously or in cycles on weekly basis (Korenblum et al., 2010). Oxidizing (chlorine and ozone), or non-oxidizing biocides (quaternary ammonium salts, aldehydes, and tetrakis -hydroxymethyl- phosphonium sulfate-THPS) are commonly applied (Gieg et al., 2011). However, this practice eventually leads to the selection of biocide-resistant microbes (Kahrilas et al., 2015).

The microbial diversity in high-temperature reservoirs includes the thermophilic genera *Thermodesulfovibrio*, *Hydrogenophilus*, *Thermodesulforhabdus*, *Pseudomonas*, *Thermovirga*, *Thermoanaerobacteraceae, Thermus, Thermodesulfobacteriaceae*, and others (Zhou et al., 2020). Members of *Tepidiphilus* have been reported in low proportion in oil reservoirs and described as a nitrate-reducing bacterium (NRB) belonging to the family Hydrogenophylaceae (Zhang X. et al., 2020). Nowadays, members of genus the *Thepidiphilus* have been reported in the produced water from thermophilic oil reservoirs, thermophilic anaerobic digesters, and hot springs from Australia, China, and India (Zhang X. et al., 2020. So far there are no reports of reference genomes for this genus in the Neotropic realm.

To date, there is limited knowledge regarding the genomic characteristics of the *Tepidiphilus* genus and its molecular capabilities to resist typical biocide treatment regimens used in the oil industry. In this work, we report and describe the genomic characteristics of a novel *Tepidiphilus* isolate found in a hyperthermophile oil well in the Orinoquia region, in Colombia, that endures the treatment with glutaraldehyde and THPS.

## Materials and Methods

### Water Samples Collection and Physicochemical Measurements

The studied oil field is in the Orinoquia region of Colombia, near the municipality of Castilla la nueva, Meta. The GPS coordinates are 3°52′46.0″N 73°37′53.0″W. The temperature in the water recovered from the oil well is estimated to be around 93°C. After sampling, the temperature of the produced water samples went down to the environmental temperature, around 26°C. The water recycling system works at environmental temperature. Before biocide-treated recycled water is reinjected into the oil well, the injection water samples were taken. About 10 L of water was taken from each sampling point and this volume was filtered on 0.45-micron diameter nitrocellulose membranes to retain the bacterial community in the filter matrix. The membranes of the filtered water samples were transferred refrigerated (4–8°C) to the laboratory for DNA extraction. Chemical analyses performed to the waters included dissolved oxygen, carbon dioxide, iron, chloride ion, and sulfate. All these tests were performed with the colorimetric kit Chemetrics (Virginia, United States): K-1910 (CO2), K-7350S (dissolved oxygen), K-6210 (iron), K-2020 (chloride ion), K-9510 (sulfate).

### Bacterial Community Metataxonomic Analysis

The DNA was extracted using the PowerSoil^®^ DNA Isolation Kit (QIAGEN) according to the manufacturer’s instructions. The DNA QC was performed by combining quantitation with PicoGreen (Invitrogen P11496) and gel electrophoresis (1% agarose) to evaluate the DNA degradation. In the produced water samples, we observed some solid particles that stacked the membrane with smaller volumes. This situation was associated with low DNA yield.

Illumina libraries were prepared and sequenced in a MiSeq (Illumina) instrument producing 300 bp paired-end reads at Macrogen Inc. (Seoul, Republic of Korea) following their recommendations. The V3-V4 hypervariable regions of bacterial and archaeal 16S rDNA gene were targeted using PCR with the primers Bakt_341F (5′-CCTACGGGNGGCWGCAG-3′) and Bakt_805R (5′-GACTACHVGGGTATCTAATCC-3′) (Herlemann et al., 2011). Amplicon libraries yielded between 128,450 and 212,074 raw reads. The amplicon reads were analyzed with the Mothur pipeline v.1.44.3 (Schloss et al., 2009). Paired-end (PE) reads were merged using Mothur’s command “make.contigs.” Sequences with homopolymers longer than 6, with ambiguous bases, or more than 466 bases in length were filtered out.

The filtered amplicon sequences were aligned to the SILVA 16S rDNA reference database (Quast et al., 2013) with the command align.seqs and only those that matched the hypervariable regions V3/V4 were retained. Next, VSEARCH (Rognes et al., 2016) was used to detect chimeric sequences. Non-bacterial lineages were removed with the command remove.lineage (chloroplasts, mitochondria, and eukaryotes). Read clustering to operational taxonomic units was performed with the subroutine “dist.seqs” at a distance limit of 0.03. Library size for each sample was normalized with the “totalgroup” method. Rare (supported by < 3 reads) OTUs were removed for downstream analyzes. The phylogenetic classification was carried out with the RDP classifier tool (bootstrap threshold set to 80) (Wang et al., 2007) and the SILVA v132 16S database. Microbial richness and diversity and indices were calculated with the R (R Development Core Team, 2018) packages Phyloseq (McMurdie and Holmes, 2013), Vegan (Oksanen et al., 2020), and Microbiome (Lahti and Shetty, 2012–2019).

### Shotgun Metagenome Analysis

For the shotgun metagenome sequencing experiment, a set of 4 DNA samples (obtained from 4 independent filters) were pooled and concentrated with Amicon Ultra Centrifugal Filters (Millipore) of each type of water: injection water-IW or produced water-PW. With each pool, one WGS library was prepared and sequenced at Macrogen Korea in one Illumina Novaseq 6000 instrument producing 150 bases paired end reads. The shotgun library was prepared with the kit TruSeq Nano DNA Kit (Illumina). Fragmentation was performed with sonication. Around 100 ng of metagenomic DNA was used for the library preparation.

The produced water WGS library yielded 77,796,614 PE reads, while the injection water library yielded 84,810,002. The reads were cleaned with CUTADAPT software removing adapters and poor-quality reads (<Q30) with flags -j 20 -q 30 -m 70 –max-n 0 (v 2.10) (Martin, 2011). Reads that were shorter than 70 bases or singletons were excluded for further analysis.

Clean reads and scaffolds were classified with MEGAN software v.6.19.9 (Huson et al., 2007) comparing the sequences using DIAMOND v2.0.11.149 (Buchfink et al., 2014) against the NCBI’s NR (Agarwala et al., 2018) protein database (February 2021). The shotgun metagenome assembly was performed with MetaSPADES version v3.14.1 (flags -t 40 -m 160) testing Kmer lengths (-k) of 21, 33, 55, 77, and 99 bases (Nurk et al., 2017).

### Injection Water Dominant *Tepidiphilus* Genome Analysis

Shotgun metagenome-assembled scaffolds were analyzed based on their nucleotide lengths and sequencing depths calculated by the assembler SPADES. Taxonomical assignment for each scaffold was obtained from MEGAN used with default settings.

The binning of the scaffolds into individual genomes was performed with VAMB version 3.0.2 (Nissen et al., 2021) with default parameters. The dominant *Tepidiphilus* genome was binned in a group of 23 scaffolds, 10 of them had nucleotide lengths below 1,000 bases, which were removed for subsequent genomic analysis. The 13 remaining scaffolds set summed 2,237,770 bp. Next, these scaffolds were submitted for genome annotation (CDSs prediction) to the DFAST web server^1^ (Tanizawa et al., 2016) activating the FastANI (Jain et al., 2018) taxonomic analysis option as well as the CHECKM v1.1.3 (Parks et al., 2015) completeness analysis. Taxonomic analysis using the digital DNA-DNA hybridization approach was performed in the TYGS website^2^ (Meier-Kolthoff and Göker, 2019). Predicted putative peptides were submitted for annotation at the KEGG database using the KAAS tool (Moriya et al., 2007) using the BBH method and the GHOSTX algorithm.

For the *Tepidiphilus* genome mixture analysis, shotgun reads of the injection and produced waters were mapped to the IW dominant *Tepidiphilus* genome using bowtie2 v. 2.4.1 (Langmead and Salzberg, 2012). The resulting SAM files were processed to obtain sorted BAM using SAMTOOLS v. 1.10 (Li et al., 2009) and variants were called with BCFTOOLS v. 1.10.2 (Li, 2011) filtering called variant with a minimum quality of 30.

The relative abundance of the predicted CDSs was calculated with the program KALLISTO 1.10.2 (Bray et al., 2016) which normalizes the library counts to each reference sequence using the TPM measurement. The fold change for each CDS was calculated with the formula Y/X – 1; being X the TPM counts for the PW shotgun reads, and Y the IW TPM counts for each CDS.

To confirm the genomic position of the CDSs with the highest fold changes, the annotated genomes were load into the ARTEMIS (Carver et al., 2012) genome browser for manual inspection and curation of the enriched CDSs. The intergenic regions were counted with ARTEMIS selecting and extracting the respective regions of interest.

### Orthologous Gene and Phylogenomic Analysis

Orthologous gene analysis was performed with SONICPARANOID program v1.3 (Cosentino and Iwasaki, 2019), using the respective predicted peptides as queries. Single and multicopy orthologous groups were detected in the *Tepidiphilus* genomes (GenBank assembly accession): *T. succinatimandens* (GCA_006503695.1), *T. thermophilus* (GCA_001418245.1), *T. margaritifer* (GCA_000425565.1), *T.* sp. J10 (GCA_006980785.1), *T.* sp. J18 (GCA_006980705.1), *T. baoligensis* B18 (GCA_012911495.1).

The above-mentioned six reference *Tepidiphilus* genomes were annotated with DFAST in the same conditions already described elsewhere.

For the phylogenomic analysis 26, single copy coding genes, commonly selected for taxonomic purposes in proteobacteria, were used: *gyrB*, *infC*, *rplA*, *rplB*, *rplC*, *rplD*, *rplE*, *rplF*, *rplK*, *rplL*, *rplM*, *rplN*, *rplO*, *rplP*, *rplT*, *rpmA*, *rpoB*, *rpsC*, *rpsE*, *rpsG*, *rpsH*, *rpsK*, *rpsL*, *rpsM*, *rpsS*, and *secY*.

A Maximum-likelihood (ML) tree was calculated with IQTREE2 v. 2.1.32 (Minh et al., 2020). Orthologous CDSs were extracted from each genome. Then, an individual alignment (one per gene) was performed using MAFFT (version v7.475) (Katoh and Standley, 2013) with parameters –inputorder –adjustdirection –anysymbol –auto. All the aligned genes in fasta format were concatenated to form a single aligned nucleotide matrix. The alignment matrix comprises 19002 sites of 9 taxa, seven *Tepidiphilus* and two outgroups, *Azoarcus communis* (GCA_003111645.1) and *Hydrogenophilus thermoluteolus* (GCA_003574215.1). The matrix was organized in two partitions with different substitutions models selected according to BIC (Bayesian Information Criterion) (Lanfear et al., 2014); as follows:

GTR + F + R2: gyrB + infC + rplE + rplL + rpoB + rpsG + rpsL, and TVM + F + I: rplA + rplB + rplC + rplD + rplF + rplK + rplM+ rplN + rplO + rplP + rplT + rpmA + rpsC + rpsE + rpsH + rpsK+ rpsM + rpsS + secY.

One thousand pseudo replicates of SH- aLRT and ultra-fast bootstrap were performed, and consensus tree generated was edited in FigTree v1.4.4.^3^ Maximum likelihood total tree length was 1.2276.

### Statistical Analysis and Graph Preparation

Statistical tests and graphical representation of data were performed with R version 4.0.4 (R Development Core Team, 2018) and R studio Version 1.2.1335. Additional packages used were: ggplot2, dplyr, ggExtra, and tidyverse.

### Next Generation Sequencing Data and Assembly Accession Numbers

Metagenome WGS raw sequences were deposited in the SRA NCBI database under the bioproject access number PRJNA727370 (SRR14424469 and SRR14424468).

Metataxonomic 16S V3-V4 amplicon sequences were deposited at the SRA database under the bioproject access number PRJNA727592 (SRR12532400, SRR12532399, SRR12532398, and SRR12532397). *Tepidiphilus* sp. UdeAICP_D1 MAG (Metagenome assembled genome) was deposited in GenBank under the accession number JAIQIL000000000. Genome and annotation in GFF format were included as Supplementary Material.

## Results

### Experiment Design and Geochemical Characterization of the Oilfield

The samples were collected in June 2020 in an oil field located in the Orinoquia region of Colombia in the Municipality of Castilla la Nueva, Meta. The reservoir has been flooded for 3 years and the produced water was taken directly from the system of crude treatment that recovers the oil/water mixture from the reservoir. The injection water was taken at the exit of the water treatment system that includes the sequential addition of glutaraldehyde and tetrakis hydroxymethyl phosphonium sulfate (THPS). The *in situ* reservoir temperature is around 93°C. The physical properties of the samples are described in Supplementary Table 1. The pH values were close to the neutral ranging from 6.9 to 7.5 for produced water (PW) and from 6.7 and 7.1 for injection water (IW). The dissolved oxygen concentration indicates an anaerobic condition in both samples, with IW having a lower oxygen concentration. Both water samples have low salinity according to the ionic constituents.

### Microbial Community Structure Based on 16S Amplicon Sequencing

**FIGURE 1:**
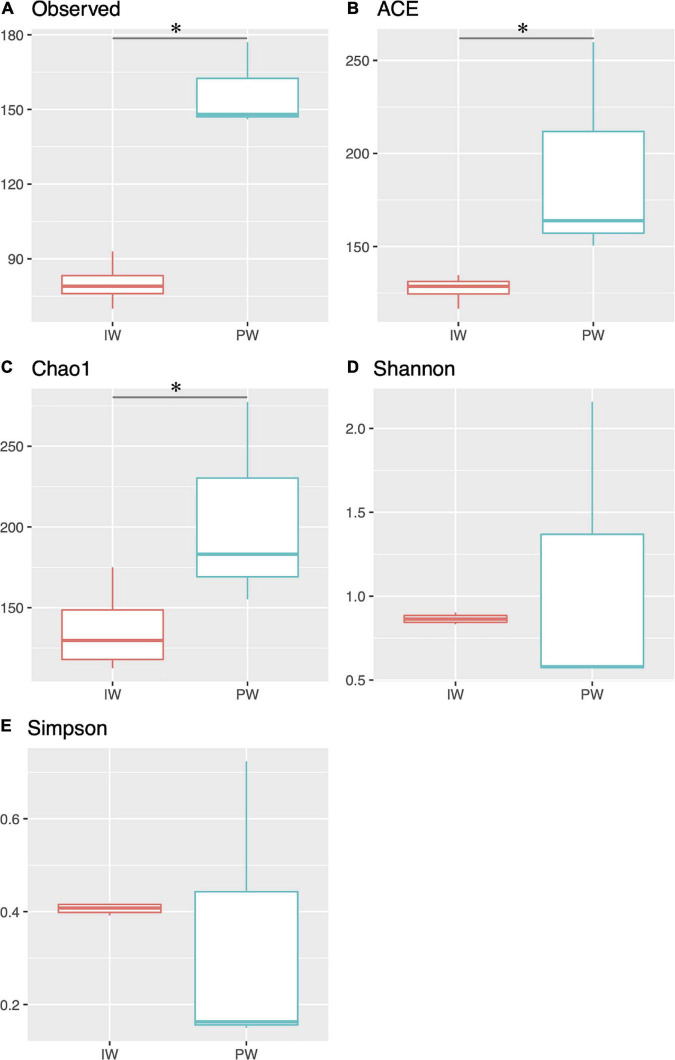
Alpha diversity analysis. Box plot comparing richness and diversity indices of the injection water (IW) and produced water (PW). **(A)** Observed OTUs. **(B)** Ace index. **(C)** Chao1 index. **(D)** Shannon Index. **(E)** Simpson Index. Asterix denotes statistical significance in the compared groups (*p* < 0.05).

**FIGURE 2:**
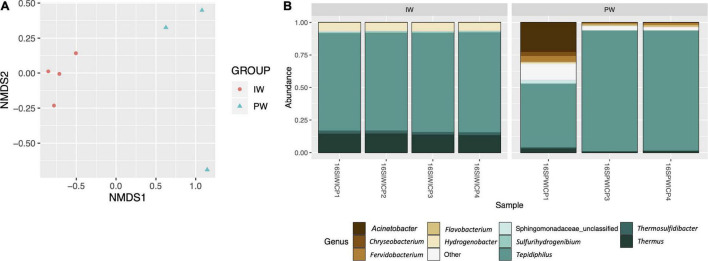
**(A)** Beta diversity analysis. NMDS ordination analysis comparing the structure of the microbial communities of the injection (IW) and produced waters (Adonis test *p* < 0.05). **(B)** Bacterial genus frequencies. Stacked histogram depicting the normalized relative abundance of the top 10 most frequent bacterial genera detected in the metataxonomic analysis of the injection (IW) and produced waters (PW).

The MiSeq amplicon sequencing experiment yielded between 128,450 and 212,074 read pairs for all tested samples. After filtering short/low-quality reads and chimeras, an average of 42,291 sequences per library was kept for further analysis (Supplementary Table 2). We excluded the replicate 16SPWICP2 due to aberrant results in both richness indices and taxonomic results. The sequences were assigned to 410 OTUs with a nucleotide identity threshold of 97%. The alpha diversity indices showed a higher richness in the PW samples (Figure 1). This observation was supported by the Kruskal-Wallis test (*p* < 0.05) in the ACE, Chao1, and observed OTUs indices (Figure 1). Shannon and Simpson diversity indices didn’t show any statistical significance (Figures 1D,E). The Good’s coverage values were ≥ 99.8% for all samples. The NMDS ordination revealed a clear separation in the IW and PW samples. The independent clustering of the different microbial communities present in the IW and PW was supported by the Adonis test (*p* < 0.05) (Figure 2A).

Most of the sequences (97.8%) were effectively classified in the genus category. A total of 26 bacterial phyla were identified. Proteobacteria (75.7% ± 0.87 IW and 90.9% ± 5.34 PW), Deinococcus-Thermus (14.06% ± 0.65 IW and 1.77% ± 1.28), and Aquificae (7.48% ± 0.19 IW and 1.11% ± 0.46) were the dominant phyla accounting for more than 90% of the whole bacterial community in both samples. In the produced water, the phyla Thermotogae (2.5%) and Bacteroidetes (2.26%) were in higher proportions (Supplementary Figure 1). At the genus level, a total of 218 taxa were classified (Figure 2B). *Tepidiphilus*, belonging to Hydrogenophilalia class, was the dominant genus in both samples, 75.6% ± 0.9 and 77.6% ± 25.06 IW and PW, respectively. *Thermus* and *Hydrogenobacter* were, respectively, the subdominant genera in the IW and PW samples accounting for 14.04% ± 0.63 and 6.38% ± 0.16.

**FIGURE 3:**
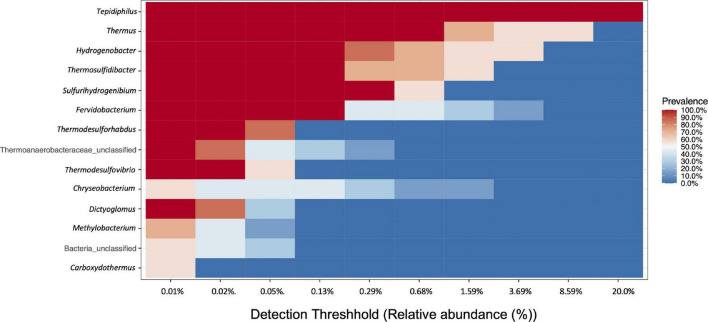
Core community analysis. Heat map representing the genera with prevalence in both water samples (IW and PW). In the *X*-axis is represented the prevalence of each genus with different detection thresholds.

A core community of 9 bacterial OTUs was detected for the IW and PW representing between 48.5 and 92.2% of all assigned sequences. This core community consisted of *Tephidiphilus*, *Thermus*, *Thermosulfidibacter*, *Hydrogenobacter*, *Thermodesulfovibrio*, *Thermodesulforhabdus*, *Sulfurihydrogenibium*, *Fervidobacterium*, and *Dictyoglomus* (Figure 3).

### Genome Analysis of the Dominant Microorganism in the Injection Water

Microbes selected in the injection waters show a resilient phenotype to the biocides THPs and glutaraldehyde applied in the oil reservoir recycled water. To gain insights into the genome structure of the dominant *Tepidiphilus* of the injection water, complementary genomic/metagenomic analyses were performed.

**FIGURE 4:**
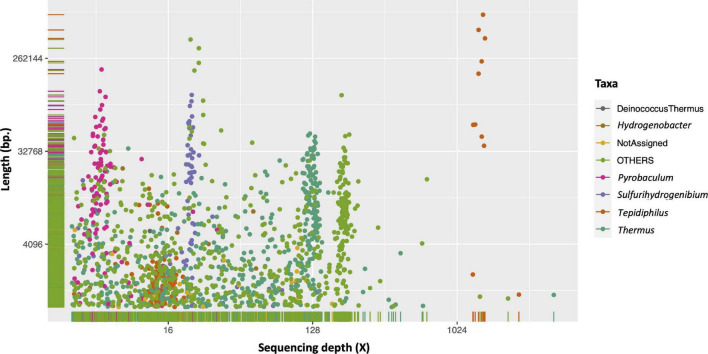
Injection water metagenome analysis of scaffold depth, length, and taxonomy. The scaffold average sequencing depth (Depth, *X*-axis) and nucleotide size (Length, *Y*-axis) are plotted using log2 transformation. The color scheme represents the respective taxa assignment for each scaffold based on the MEGAN results.

The metagenome of the injection water was *de novo* assembled, yielding 38,342,519 total bases contained in 38,581 scaffolds. Analysis of the nucleotide length, average sequencing depth, and the taxonomic assignation of each scaffold show that sequences of the same microorganism tend to cluster in a narrow sequencing depth range (Figure 4). Moreover, the taxonomic data (color of the dots) supports the related taxonomic origin. In concordance with the previous metataxonomic and taxonomic analysis of the shotgun reads, we observed a clear dominance of *Tepidiphilus* (orange dots), which exhibits the longest scaffold assembled (695,641 bases). It is also evident that two clusters of *Tepidiphilus* scaffolds were present, one that exceeds the 1,000X average sequencing depth and another at around 16X with lower nucleotide length counts.

Other relevant microorganisms observed in this metagenome are *Thermus* and *Sulfurihydrogenibium*, and the Archaea *Pyrobaculum*.

The genome of the IW dominant *Tepidiphilus*, from now on termed UdeAICP_D1, was isolated using the software VAMP. To achieve this, the shotgun reads were mapped to the metagenome-assembled scaffolds, and then the reads that link different scaffolds allow the clustering of sequences that comes from the same chromosome. Scaffolds shorter than 1,000 bases were excluded from the subsequent genomic analysis. The isolated genome comprises 2,237,527 bp in 13 scaffolds. The assembly N50 value was 494,759 bases, and it exhibits a GC content of 66%. Genome annotation identified 2,108 CDS sequences along with 2 rRNA genes, 51 tRNAs genes, and one CRISPR gene. The average protein length was 333 amino acids, and the coding ratio was 94.1%. The gap ratio was estimated to be 0.004469%. Genome completeness analysis using CHECKM for proteobacteria gave a 100% score and a contamination signal below 1%.

Taxonomic analysis using the FastANI tool indicates that *Tepidiphilus* sp. UdeAICP_D1 is a species closely related to *Tepidiphilus succinatimandens* (ANI score 98.3291) and *Tepidiphilus thermophilus* (ANI score of 98.322) with a nearly equal score for both species. Digital DNA-DNA hybridization analysis classified as *Tepidiphilus* sp. UDEAICP_D1 as *T. thermophilus* with a dDDH score of 83.7%. It should be mentioned that *Tepidiphilus succinatimandens* is also above the dDDH species threshold with a dDDH 82.7%.

Taxonomic assignment confirmation was performed using a phylogenomic analysis based on 26 single copy conserved protein-coding genes. The concatenated alignment comprises 19,002 sites. *Azoarcus communis* and *Hydrogenophilus thermoluteolus* were selected as outgroups. The best evolution model (mixed) was selected according to BIC (GTR and TVM). The best maximum likelihood tree had a log-likelihood value of −49969.3879.

**FIGURE 5:**
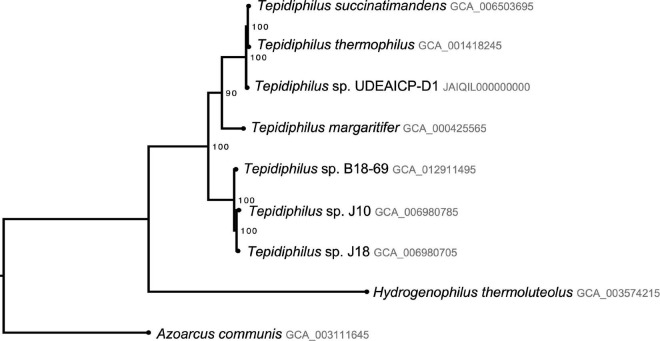
Phylogenomic analysis of the *Tepidiphilus* species based on 26 single copy conserved protein-coding genes. Maximum likelihood tree with 1,000 pseudoreplicates of ultrafast bootstrap.

The tree shows two well-supported (100% UFB) clades, one that comprises the isolates J10, J18, and B18, and another that comprises *T. succinatimandens*, *T. thermophilus*, and *Tepidiphilus* UdeAICP_D1. Furthermore, *T. margaritifer* is positioned outside both clades with slightly lower support (90%). The tree also indicates that *Tepidiphilus* sp. UdeAICP_D1 is an independent branch outside *T. succinatimandens* and *T. thermophilus* with 100% UFB support (Figure 5).

### Metabolic Potential of *Tepidiphilus*

The genome of *Tepidiphilus* UdeAICP_D1 recovered from the injected water was annotated, and key metabolic pathways were predicted with the KEGG (Kyoto Encyclopedia of Genes and Genomes) database. The annotation comparison with the *Tepidiphilus* genomes deposited in public databases identified a virtual proteome that ranged from 2,033 and 2,288 peptides. Orthologous protein analysis clustered *Tepidiphilus* proteomes into 2,370 orthologous groups. Most of the *Tepidiphilus* putative pan proteome is shared among the studied species since more than 92% of the predicted proteins are shared by at least 2 genomes. The core proteome, shared by all seven *Tepidiphilus* genomes, comprises 1,621 orthologous protein groups, of which 1,596 are single-copy genes. All *Tepidiphilus* isolates have unique proteins, with no detected orthologous, with counts that ranged between 58 (*Tepidiphilus thermophilus*) and 183 (*Tepidiphilus succinatimandens*). *Tepidiphilus* UdeAICP_D1 has 90 specific putative proteins. Singleton protein count (with no orthologous) in the other species were *T. baoligensis* B18, *n* = 156; *T.* sp. J18, *n* = 170; *T.* sp. J10, *n* = 75; and *T. margaritifer, n* = 149 (Supplementary Table 3).

The *Tepidiphilus* sp. UdeAICP_D1 putative proteome functional annotation showed 51 complete modules and putative proteins that are part of 227 KEGG pathways. Moreover, *Tepidiphilus* sp. UdeAICP_D1 encodes the enzymes required for the biosynthesis of the amino acids: threonine, cysteine, isoleucine, lysine, ornithine, arginine, and proline. The carbon fixation was related to acetate production. Enzymes involved in the assimilatory sulfate reduction (CysND, CysC, CysH, and CysJI) and the denitrification pathway were also detected.

Several *Tepidiphilus* sp. UdeAICP_D1 genes could be involved in its biocide and stress-resistant phenotype. Five efflux pumps modules were identified: MexAB-OprM, MexJK-OprM, MexXY-OprM, AcrEF-TolC, and MdtEF-TolC. These efflux pumps are present in all seven *Tepidiphilus* genomes. Additionally, five type II toxin-antitoxin (TA) systems were found in *Tepidiphilus* chromosomes: YhaV-PrlF, YoeB-YefM, ParE-ParD, HigB-HigA, and FitB-FitA. Other genes related to the response to stress were annotated in this genome, namely PhoR-PhoB (phosphate starvation response), EnvZ-OmpR (osmotic stress response), CusS-CusR (copper tolerance), and QseC-QseB (quorum sensing regulator B and C related with the motility).

### *Tepidiphilus* Genome Biocide Selection

The metagenomic analysis showed that *Tepidiphilus* dominated injection and produced water systems, although the injection water was a more restrictive environment due to the biocide treatment. To gain insights into the possible mixture of different *Tepidiphilus* genomes present in both samples and the selection process exerted by the biocide regime, a mapping, and single nucleotide variant (SNV) detection analysis was performed. To this end, the isolated genome of the *Tepidiphilus* sp. UDEAICP_D1 was used as a reference, and the shotgun metagenomic reads of both IW and PW were mapped to identify nucleotide variants that reflect the genomic diversity of this bacteria within the studied industrial waters.

**FIGURE 6:**
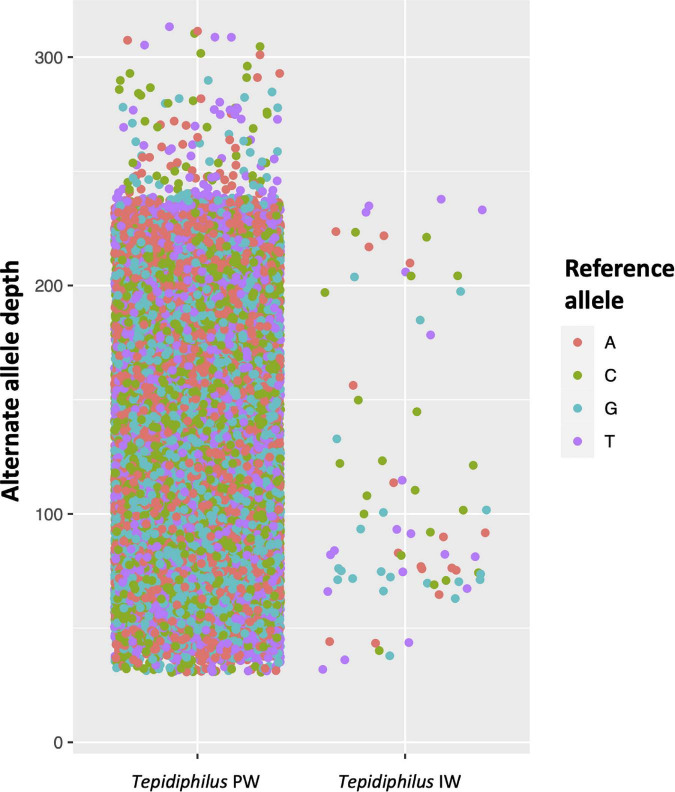
Genomic variations among *Tepidiphilus* genomes present within the injection (IW) and produced waters (PW). Detection of single nucleotide variants -SNVs- in the *Tepidiphilus* genomes. Each dot represents high-quality SNVs detected in the shotgun reads of the PW and IW. The respective reference base (Reference Allele) is represented as different colors. The *Y*-axis depicts the read depth for each SNV observed. In the IW, 95 alternative bases were detected while in the PW 32,567 high-quality alternate alleles were encountered.

The genome mapping analysis with IW reads identified only 95 well-supported (≥Q30) positions with an alternate nucleotide. This result indicates that the isolated genome scaffolds of *Tepidiphilus* sp. UDEAICP_D1 represents an accurate genome model and is somehow pure due to the low number of genomic variations (>0.005%). However, the PW read mapping analysis shows a mixture of *Tepidiphilus* genomes since more than 32,567 SNVs were detected with very diverse coverage (Figure 6). In the PW reads, it was also possible to discern some positions with a well-supported (>Q30) third SNV.

**FIGURE 7:**
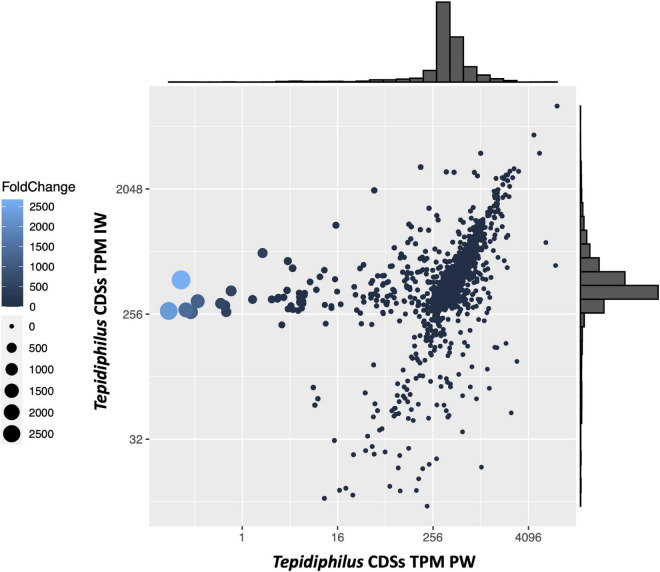
*Tepidiphilus* CDS relative abundance using TPM. The *X* and *Y*-axes represent the calculated TPM for each CDS for the PW and IW, respectively. Both axes are log2 transformed. Dot size and the lighter color are proportional to the CDS fold change: The bigger the dots or lighter the color, means higher fold change. Outer histograms represent the frequency of scaffolds in each region of the graph.

To gain insights into the genomic characteristics of the *Tepidiphilus* sp. UDEAICP_D1 genome selected in the IW, an analysis of the enriched coding regions was performed. To this end, using as reference the annotated genome of the *Tepidiphilus* sp. UDEAICP_D1, the shotgun reads of both IW and PW libraries were mapped to the annotated CDSs and then counted, normalized, and plotted (Figure 7). The normalized counts (TPMs) obtained for the PW reads are depicted in the *X*-axis, whereas in the *Y*-axis are the IW TPMs. In addition, fold-change measurements were calculated with the aim to compare the relative abundance of each CDS between both metagenomes. Most of the CDSs form a cluster above the 256 TPM values on both samples. This result indicates that most genes are shared among the different *Tepidiphilus* genomes. The interesting CDSs are those with unbalanced TPM values in both metagenomes. The CDSs sequences with values above 256 (*Y*-axis) and below the 16 value (*X*-axis) display a strong enrichment within the *Tepidiphilus* sp. UDEAICP_D1 genome.

Enriched genes in the IW *Tepidiphilus* sp. UDEAICP_D1 genome were explored using a fold-change metric. This analysis pointed to a group of six genes with the highest fold-change values, between 500 and 2,600 (Supplementary Table 4, TepidiUDEAICP_19830-80). Interestingly, these genes are organized as a cluster in the same strand and with short intergenic regions of 10 or fewer bases. Unfortunately, the annotation tools retrieve no hits for most of them. Just one gene had a weak hit with a proteobacterial Heat shock 70 kDa protein.

The following enriched group consists of four genes, also organized in a tandem structure, and located in the same strand with very short intergenic regions (<5 bp.). Their fold-change ranged between 20 and 569 (Supplementary Table 4, TepidiUDEAICP_09260-90). The annotation results obtained for these proteins indicated putative functions as ABC-2 type transporter, ABC transporter, Methionine biosynthesis protein MetW, and Glycosyltransferases group. Another group of five genes with a high fold-change was related to the two-component system NarL (Supplementary Table 4, TepidiUDEAICP_11610-50), which is sensible to nitrate/nitrite ligands. This system is absent in the *Tepidiphilus* isolates B18, J10, and J18. Finally, it was possible to identify yet another group of 7 genes organized in an operon-like structure with fold-change values that ranged between 52 and 68 (Supplementary Table 4, TepidiUDEAICP_00090-150). The annotation results for this gene cluster indicate that they are related to enzymes with functions like: Short-chain dehydrogenase reductase, reversible phosphatidyl group transfer, DNA methylase, tRNA cytidylyltransferase, and Superfamily II DNA RNA helicases.

## Discussion

The metataxonomic and metagenomic analysis performed in this work unveiled the microbial diversity present in a hyperthermophile oil reservoir of the Orinoquia region in Colombia, South America. In the oil industry, it is often common to recycle oil well water to be used again to flood the reservoir. This procedure is named secondary recovery and allows for more oil extraction. Recycled water is treated with biocides to prevent the undesired effect of the native of introduced microbiota in the oil recovery process.

The produced water samples might reflect a closer image of the native oil reservoir microbiota, whereas the injection water (recycled water with added biocides) microbiota contains indigenous biocide resilient microbes and those artificially introduced, which are also resistant to biocides, and that are capable of colonizing piping systems. As expected, our results show that the biocide regime dramatically reduces microbial richness and wipes out certain microbes in the injection water, but some bacteria endure.

The dominant bacterial genera observed as indigenous microbes in this oil reservoir has already been described in other thermophilic environments: *Tepidiphilus*, *Thermus*, *Thermosulfidibacter*, and *Hydrogenobacter*. These bacterial genera have been isolated from the produced water from oilfields that use secondary recovery by water injection with temperatures, *in situ*, above 55°C (Ren et al., 2015; Pannekens et al., 2019). However, it is still a matter of discussion if all these microorganisms are indigenous or some are introduced artificially with the injected water used for the secondary oil recovery (Vigneron et al., 2017).

The genus *Thermus* has been previously isolated from thermal environments, including oil reservoirs. This bacterium is associated with alterations of the physicochemical properties of crude petroleum and with the degradation of hydrocarbons (Nikolova and Gutierrez, 2020). *Thermosulfidibacter* is a thermophilic anaerobic bacterium initially isolated from deep-sea hydrothermal vents. In oil reservoirs, this bacterium is related to sulfur reduction (Nunoura et al., 2008). *Hydrogenobacter* is a hydrogen-oxidizing bacterium that is part of the thermophilic deep terrestrial water microbial community (Takai et al., 2001).

*Tepidiphilus* dominated all the water samples analyzed in this industrial system, injection, and produced, despite the recycling and biocide treatment. This genus has been reported in hot springs, thermophilic anaerobic digesters, and produced water from oil reservoirs (Zhang X. et al., 2020). This bacterium is described as a rod-shaped cell, gram-negative, motile by polar flagella, and capable of anaerobic growth in the presence of nitrate (Poddar et al., 2014).

Our genomic analysis demonstrated the existence of a *Tepidiphilus* microdiversity within the water recovered from the oil reservoir (produced water). After the water recycling and biocide addition, this microdiversity disappears, and only one clonal *Tepidiphilus* is selected. Due to the strong selection of this *Tepidiphilus* in the injection water, the metagenomic analysis allowed us to easily isolate and characterize its metagenome-assembled genome.

The phylogenomic analysis confirmed its genus but showed that *Tepidiphilus* sp. UdeAICP_D1 is an independent, and well-supported branch (100% UFB) outside *T. succinatimandens* and *T. thermophilus* clade. This analysis also showed very low distances between these three organisms. The ANI and dDDH approaches showed conflicting results among them and with our phylogenomic results. Nonetheless, the phylogenomic analysis is the most theoretically rigorous approach available to resolve evolutionary relationships between organisms. Adequate bacterial taxonomical methods should rely on the evolutionary relationship between the species (Parks et al., 2018). Further analysis, including more genomes, should be performed to support this finding.

When individual genomes are isolated from metagenomic data, one utmost concern arises and is related to the possibility of generating mixed/chimeric scaffolds (Meziti et al., 2021). To overcome this limitation, mapping analyses were performed to identify mixed nucleotide signals in the isolated reference genome of *Tepidiphilus* sp. UdeAICP_D1. With this strategy, we were able to spot only 95 SNVs along the whole genome. This result indicated that shotgun reads support a pure clonal genome. Additionally, the completeness and contamination analysis reported by CHECKM reported values of 100% and < 1%, respectively, confirming the quality of this metagenome-assembled genome (MAG) from the injection water. By contrast, the mapping and the CDS depth analysis revealed a complex mixture of closely related *Tepidiphilus* genomes within the produced water. Nonetheless, specific genomic blocks, encompassing certain genes, differentiate these different *Tepidiphilus* genomes. In the end, it seems that only one genome carries the necessary gene repertoire to endure and thrive in the harsh conditions imposed by physicochemical stress-induced outside the oil well.

Comparative genomic analysis revealed highly conserved genomes within the *Tepidiphilus* genus. Most of the virtual proteome is shared (92%) by at least two members. Nonetheless, all the analyzed species presented several specific CDSs. *Tepidiphilus* core proteome is organized in 1,621 orthologous groups present in all the studied species.

The dominance of *Tepidiphilus* sp. UDEAICP_D1 within the injection water might suggest that it is the most adapted microbe to the stringent conditions of this recycled water. Its genome comprises a combination of genes that help withstand biocide, metabolic and competence challenges like ABC transporters, NarL two-component systems, and TA-systems. Furthermore, *Tepidiphilus* sp. UDEAICP_D1 also encompasses genes related to toxic chemical resistance and stress response.

The microbial species present in the oil reservoirs are exposed to different types of selective pressure, such as chemical treatment (biocides) or high concentrations of lipids and heavy metals. This scenario might play a role in the prevalence of multidrug efflux transporters in the indigenous *Tepidiphilus* community. The multidrug efflux pumps MexJK-OprM, MexXY-OprM, MexAB-OprM, AcrEF-TolC, and MdtEF-TolC, were observed in all *Tepidiphilus* genomes. They belong to the resistance–nodulation–cell division (RND) superfamily. These molecular pump systems encompass three elements: an inner membrane protein component with proton motive function, a channel-forming outer membrane factor (OMF), and a periplasmic membrane fusion protein (MFP) (Delmar et al., 2014). RND complexes are commonly found in Proteobacteria and have been extensively studied in human infecting species, but the information in non-infectious environmental prokaryotes is scarce (Rahman et al., 2017).

The efflux pumps AcrEF-TolC and MdtEF-TolC have been extensively studied in *Escherichia coli.* They are associated with resistance to antibiotics like quinolones, Erythromycin, DNA intercalating agents like ethidium bromide, and anionic detergents like Sodium dodecyl sulfate SDS (Anes et al., 2015). Additionally, MdtEF-TolC is shown to protects *E. coli* cells from the toxicity of nitrosative stress during anaerobic respiration of nitrate. A similar effect could be expected in *Tepidiphilus* in the underground thermophilic anaerobic environment (Zhang Y. et al., 2011).

In general terms, RND systems confer resistance to a wide spectrum of antibiotics like aminoglycosides, fluoroquinolones, and beta-lactams. Also confer resistance to lipids, toxins, detergents, herbicides, dyes, and biocides (Venter et al., 2015; Tsutsumi et al., 2019).

Moreover, RND efflux pumps have been associated with glutaraldehyde resistance. In *Pseudomonas*, Vikram et al. (2015) found that efflux pumps contribute to glutaraldehyde endurance since RND inhibitors can potentiate glutaraldehyde activity. In *P.* aeruginosa, a famous microbe due to its wide-spectrum resistance to antimicrobial agents (Poole, 2011), four RND pumps have been described (Morita et al., 2001). Likewise, we found five of these molecular pump systems encoded in the *Tepidiphilus* genome.

The several toxin-antitoxin (TA) systems detected in the genome of *Tepidiphilus* sp. UdeAICP_D1 might contribute, as observed in other bacterial taxa, to bacterial stress responses, biofilm formation, virulence, antibiotic tolerance, and persistence bacteria formation (Zhang S.-P. et al., 2020). The TA system consists of pairs of genes located in one operon: a toxic protein, and a labile antitoxin. These systems can be activated in response to stress conditions and are crucial to compete and thrive in harsh conditions inhibiting translation or DNA replication of bacterial competitors (Harms et al., 2018). YhaV and YoeB are ribonuclease toxins of the RelE family. Both can arrest cell growth and are inhibited by their respective antitoxin PrlF and yefM. This TA system was initially described in *E. coli* (Schmidt et al., 2007), yet RelE toxins have been found on other Proteobacteria as well as in Firmicutes (Leplae et al., 2011). YefM-YoeB is one of the most frequent type II TA systems detected in pathogenic bacteria. This system is activated during thermal stress and confers oxidative resistance, biofilm formation, and antibiotic resistance (Ma et al., 2021). In the ParE-ParD TA system described in the Enterobacteriaceae family, ParE is a gyrase inhibitor that blocks DNA replication and provides antibiotic and heat tolerance. *Tepidiphilus* also carries a gene coding for the HigB mRNA endonuclease toxin which is inhibited by its antitoxin HigA. This TA system belongs to the HigA superfamily, which is found in Proteobacteria, Firmicutes, and Cyanobacteria (Leplae et al., 2011). FitB-FitA is another TA system that belongs to the VapB superfamily and that it is present in Bacteria and Archaea (Senissar et al., 2017). FitA, the antitoxin, is a DNA-binding protein with a putative ribbon-helix-helix (RHH) motif that neutralizes the toxin FitB through protein–protein interaction (Senissar et al., 2017).

Previous studies have reported that the ParE-ParD system stimulates bacterial growth at high temperatures and the biofilm formation in *E. coli* (Kamruzzaman and Iredell, 2019). ParE is a toxin that inhibits DNA gyrase inhibiting chromosome replication (Dalton and Crosson, 2010).

## Conclusion

In conclusion, *Tepidiphilus* displays several type II TA systems and RND efflux pumps. In combination, both improve bacterial fitness and extrude toxic compounds that might be present in the oil reservoir or related to the biocides regime. Five type II TA systems were annotated in all *Tepidiphilus* genomes. These systems are associated with biofilm formation and stress response (Wang and Wood, 2011). It is demonstrated that bacterial communities organized under biofilm structures show higher resilience to biocide treatment (Mah and O’Toole, 2001). Additionally, five RND efflux pumps are carried by the *Tepidiphilus* genomes, and these systems are associated with the bacterial ability to withstand glutaraldehyde treatment (Vikram et al., 2015).

We consider that there are, at least, two potential areas that should be addressed shortly for *Tepidiphilus*. One involves the revision of the taxonomy of the members of the genus, and the other is related to targeted gene annotation. Sequencing of more *Tepidiphilus* genomes is necessary to validate, through phylogenomic analyses, the actual species scheme. On the other hand, *in vitro* biochemical analysis should be performed in order to gain insights into the function of the highly enriched genes found in *Tepidiphilus* sp. UDEAICP_D1. The current annotation programs and databases fail to assign a function to their respective gene products. The annotation of these genes will help to clarify not only its role in *Tepidiphilus* fitness, but also in other Proteobacterial species.

## Data Availability Statement

The datasets presented in this study can be found in the NCBI SRA under accession numbers PRJNA727370 and PRJNA727592 (https://www.ncbi.nlm.nih.gov/). The MAG Tepidiphilus genome is also available under accession JAIQIL000000000.

## Author Contributions

KB: wetlab experiments, DNA extraction and QC, bioinformatics and statistical analyses, and manuscript writing and review. JN: wet lab experiment design, sample collection, and processing, manuscript review. JA: wet lab experiment design and manuscript review. RJ-P: wet lab experiment design, sample collection and processing, and manuscript review. FC: bioinformatics and statistical analyses, and manuscript writing and review. JFA: project design and coordination, analysis planning, bioinformatics and statistical analyses, and manuscript writing and review. All authors contributed to the article and approved the submitted version.

## Conflict of Interest

The authors declare that the research was conducted in the absence of any commercial or financial relationships that could be construed as a potential conflict of interest.

## Publisher’s Note

All claims expressed in this article are solely those of the authors and do not necessarily represent those of their affiliated organizations, or those of the publisher, the editors and the reviewers. Any product that may be evaluated in this article, or claim that may be made by its manufacturer, is not guaranteed or endorsed by the publisher.

## Footnotes

1
https://dfast.ddbj.nig.ac.jp/

2
https://tygs.dsmz.de/

3
http://tree.bio.ed.ac.uk/software/figtree/
