# Supplementary material for: Metagenomic Analysis of Biocide-Treated Neotropical Oil Reservoir Water Unveils Microdiversity of Thermophile Tepidiphilus
Source: Front Microbiol. 2021 Nov 1;12:741555. doi: 10.3389/fmicb.2021.741555 (PMC8591294; doi:10.3389/fmicb.2021.741555)
Supplement: Supplementary file 1 [file Data_Sheet_1.ZIP › Supplementary_Material.docx]

Supplementary Material

# Supplementary Tables

**Supplementary** **Table 1.** Physicochemical characteristic of the samples

|  | **Average pH** | **O_2_ (ppb)** | **CO_2_ (mg/L)** | **Fe++ (mg/L)** | **SO4-- (mg/L)** | **Cl- (mg/L)** | **Total Alkalinity (mg CaCo3/L)** |
| --- | --- | --- | --- | --- | --- | --- | --- |
| Produced water | 7,18 | 100 | <10 | 0,13 | 12,9 | 86 | 64,5 |
| Injected water | 6,89 | 12,50 | 11,22 | 0,39 | 9,93 | 99,67 | 63,44 |

**Supplementary Table 2. Alpha diversity indices**

| Sample code | Observed | Chao1 | ACE | Shannon | Simpson |
| --- | --- | --- | --- | --- | --- |
| 16SIWICP1 | 93 | 112.5 | 116.6272 | 0.9027005 | 0.4174164 |
| 16SIWICP2 | 80 | 119.6667 | 130.0928 | 0.8793291 | 0.4148741 |
| 16SIWICP3 | 78 | 139.75 | 134.5637 | 0.8475916 | 0.4006637 |
| 16SIWICP4 | 70 | 175 | 127.0993 | 0.8323593 | 0.3915809 |
| 16SPWICP1 | 146 | 155.1667 | 150.5235 | 2.1583212 | 0.7235538 |
| 16SPWICP3 | 148 | 183.1 | 163.8481 | 0.5706055 | 0.14995 |
| 16SPWICP4 | 177 | 277.5 | 259.7529 | 0.5795228 | 0.1626556 |

## Supplementary Figures

**Supplementary Figure 1.** **Bacterial phyla frequencies.** Stacked histogram of the top ten most frequent bacterial phyla detected in the metataxonomic analysis of the injection (IW) and production waters (PW).
